# Identification of Leukocyte Surface P2X7 as a Biomarker Associated with Alzheimer’s Disease

**DOI:** 10.3390/ijms23147867

**Published:** 2022-07-17

**Authors:** Yihan Li, Xin Huang, Christopher Fowler, Yen Y. Lim, Simon M. Laws, Noel Faux, James D. Doecke, Brett Trounson, Kelly Pertile, Rebecca Rumble, Vincent Doré, Victor L. Villemagne, Christopher C. Rowe, James S. Wiley, Paul Maruff, Colin L. Masters, Ben J. Gu

**Affiliations:** 1 The Florey Institute, The University of Melbourne, 30 Royal Parade, Parkville, VIC 3052, Australia; yihan.li@florey.edu.au (Y.L.); xin.huang@florey.edu.au (X.H.); christopher.fowler@florey.edu.au (C.F.); btrounson@unimelb.edu.au (B.T.); kelly.pertile@florey.edu.au (K.P.); rebecca.rumble@florey.edu.au (R.R.); jwiley@unimelb.edu.au (J.S.W.); pmaruff@unimelb.edu.au (P.M.); c.masters@unimelb.edu.au (C.L.M.); 2 School of Psychological Sciences, Turner Institute for Brain and Mental Health, Monash University, Clayton, VIC 3168, Australia; yenying.lim@monash.edu (Y.Y.L.); vincent.dore@csiro.au (V.D.); 3 School of Medical and Health Sciences, Edith Cowan University, 270 Joondalup Drive, Joondalup, WA 6027, Australia; s.laws@ecu.edu.au; 4 Melbourne Data Analytics Platform, Petascale Campus Initiative, The University of Melbourne, 21 Bedford St., North Melbourne, VIC 3051, Australia; nfaux@unimelb.edu.au; 5 The Australian e-Health Research Centre, CSIRO, Brisbane, QLD 4029, Australia; james.doecke@csiro.au; 6 Department of Molecular Imaging & Therapy, Austin Health, Melbourne, VIC 3084, Australia; victor.villemagne@pitt.edu (V.L.V.); crowe@unimelb.edu.au (C.C.R.); 7 Department of Medicine, The University of Melbourne, Melbourne, VIC 3084, Australia; 8 Department of Psychiatry, University of Pittsburgh, Pittsburgh, PA 15260, USA; 9 CogState Ltd., Melbourne, VIC 3001, Australia; 10 National Clinical Research Center for Aging and Medicine, Huashan Hospital, Fudan University, Shanghai 200040, China

**Keywords:** myeloid cells, purinergic receptors, episodic memory, the Preclinical Alzheimer’s Cognitive Composite (PACC), CSF T-tau, CSF Aβ_1-42_, CSF P-tau181P, brain atrophy

## Abstract

Alzheimer’s disease (AD) has shown altered immune responses in the periphery. We studied P2X7 (a proinflammatory receptor and a scavenger receptor) and two integrins, CD11b and CD11c, on the surface of circulating leukocytes and analysed their associations with Aβ-PET, brain atrophy, neuropsychological assessments, and cerebrospinal fluid (CSF) biomarkers. Total 287 age-matched, sex-balanced participants were recruited in a discovery cohort and two validation cohorts through the AIBL study and studied using tri-colour flow cytometry. Our results demonstrated reduced expressions of P2X7, CD11b, and CD11c on leukocytes, particularly monocytes, in Aβ +ve cases compared with Aβ −ve controls. P2X7 and integrin downregulation was observed at pre-clinical stage of AD and stayed low throughout disease course. We further constructed a polygenic risk score (PRS) model based on 12 *P2RX7* risk alleles to assess the genetic impact on P2X7 function in AIBL and ADNI cohorts. No significant association was identified between the *P2RX7* gene and AD, indicating that P2X7 downregulation in AD is likely caused by environmental changes rather than genetic factors. In conclusion, the downregulation of P2X7 and integrins at pre-clinical stage of AD indicates altered pro-inflammatory responses, phagocytic functions, and migrating capabilities of circulating monocytes in early AD pathogenesis. Our study not only improves our understanding of peripheral immune involvement in early stage of AD but also provides more insights into novel biomarker development, diagnosis, and prognosis of AD.

## 1. Introduction

Alzheimer’s disease (AD) is the leading form of dementia, characterised by impairment in learning, memory, and performance of daily activities [1]. The cardinal pathological hallmarks of AD include neurofibrillary tangles and beta-amyloid (Aβ) plaques in the brain [1]. The imbalanced production and clearance of neurotoxic Aβ peptides is a very early, initiating factor in AD [2,3]. Pathological Aβ accumulation, especially Aβ_1-40_ and Aβ_1-42_, stimulates microglial and astrocytic activation and pro-inflammatory responses to clear Aβ [2,3]. These gradually altered immune cells and inflammatory responses drive disease progression before AD symptoms are observed, as supported by genetic studies [1,3,4]. These early biological changes can occur up to 30 years before the appearance of AD clinical symptoms [5,6], making this preclinical stage of the disease ideal for both understanding and potentially treating AD. Our research goal is to understand immune involvement in preclinical and prodromal AD, improve the clinical pathological model of AD, and further provide potential insights into biomarker discovery and therapeutic development.

Recent genome-wide association studies (GWAS) of AD have discovered over 130 genomic loci associated with AD, demonstrating their roles in microglial phagocytosis of Aβ, particularly phagocytic receptors and endolysosomal network of the innate immune system [4,7]. In addition to the interaction between Aβ, tau, and glial cells in AD brain, new evidence demonstrates the crosstalk between the central nervous system (CNS) and the peripheral blood and immune system in AD. As AD progresses, increased vascular permeability of blood-brain barriers (BBB) allows for a selective entry of peripheral immune cells into the CNS [8]. Peripheral monocytes and T lymphocytes are suggested to infiltrate into the CNS, modulate inflammatory responses, and promote microglial phagocytosis of Aβ in mice models of AD [9]. Cerebral Aβ can be transported into the peripheral pool via BBB, cerebrospinal fluid (CSF), arachnoid villi, and glymphatic-lymphatic system [10]. Nearly half of cerebral Aβ can be exported into the peripheral circulation to be cleared by enzymes, immune cells, tissues, or organs [4]. The genomic studies of AD have demonstrated monocyte-specific enrichment of AD risk variants, indicating that AD risk variants may locate in the regulatory regions to modulate monocytic function [4]. Our group also reported the first human evidence that the basal phagocytic ability of monocytes was associated positively with cerebral Aβ burden in AD patients [11]. Hyperactivation of monocytes were observed in the prodromal stage of AD, including increased chemotaxis, free radical production, and cytokine production [12]. These findings all suggest that the phagocytic function and pro-inflammatory responses of peripheral monocytes are associated with Aβ burden during AD progression.

This study aimed at understanding the peripheral immune involvement in AD, regarding the phagocytic function and pro-inflammatory responses of innate immune cells. Our group has studied P2X purinoceptor 7 (P2X7) for decades, which is a dual-functional purinergic receptor that acts as an ATP-induced pro-inflammatory ion channel and a scavenger receptor responsible for phagocytosing apoptotic cells and debris [13]. Additionally, recent AD GWAS has nominated many microglial-specific risk genes, including CD11b (integrin alpha M, *ITGAM*, or α_M_) and CD11c (integrin alpha X, *ITGAX*, or α_X_), which are integrins responsible for phagocytosis mediated by complement system and the recruitment and migration of monocytes [4]. Given that AD GWAS and functional studies all indicate the role of phagocytosis in AD, the performance of P2X7, CD11b, and CD11c on professional phagocytes was the primary aim of this study, including monocytes and neutrophils. The role of adaptive immune system in AD has also been documented [4], so T lymphocytes, B lymphocytes, and natural killer (NK) cells were also cell types of interest. Given that Aβ plays a predominant role in the preclinical, prodromal, and dementia stages of the disease [14] and positron emission tomography (PET)-Aβ imaging (measured by Centiloid [CL]) provides the most accurate diagnosis of AD [15], the performance of P2X7, CD11b, and CD11c were compared by PET-Aβ status (−ve: ≤25 CL/+ve: >25 CL), followed by further comparisons by symptoms(cognitively normal [CN] −ve, CN +ve, mild cognitive impairment [MCI] −ve, and dementia [MCI +ve and AD]). In addition to PET-Aβ imaging, brain atrophy assessed by magnetic resonance imaging (MRI), cognitive decline assessed by neuropsychological scores (episodic memory [EM] and the Preclinical Alzheimer’s Cognitive Composite [PACC]), and CSF measurements of Aβ_1-42_, total tau (T-tau), and tau phosphorylated at threonine 181 (P-tau181P) all reflect the natural history of AD in the three major stages [6]. Therefore, we further evaluated the associations between our receptors of interests, MRI volumetrics, EM, PACC, and CSF biomarkers to study their roles in disease course.

## 2. Results

### 2.1. P2X7 Downregulation in AD

#### 2.1.1. Reduced Leukocyte Surface P2X7 Expression in Aβ +Ve Cases in Discovery Cohort

**Figure 1:**
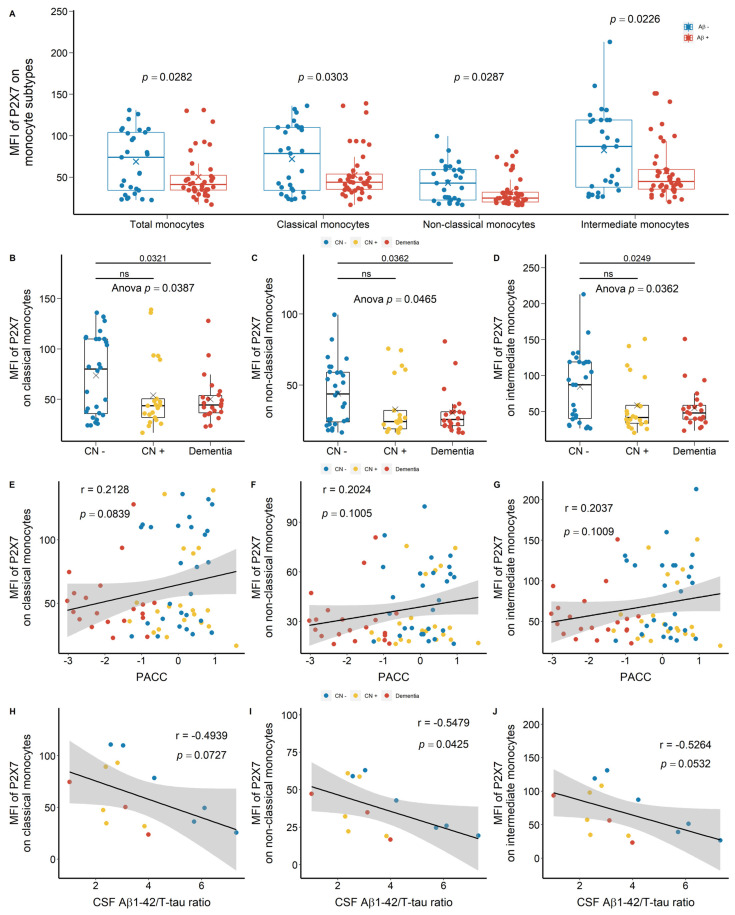
P2X7 expressions on monocytes in the discovery cohort. (**A**) P2X7 expressions on total monocytes (*left 1*), CD14^+^CD16^−^ classical monocytes (*left 2*), CD14^dim^CD16^+^ non-classical monocytes (*right 2*), and CD14^+^CD16^+^ intermediate monocytes (*right 1*) between Aβ −ve controls (Aβ−) and Aβ +ve cases (Aβ+). Bar graphs illustrated the boxplot distribution of individual measurements with “x” denoting the mean. Two group comparison was determined by *t*-test. (**B**–**D**) P2X7 expressions on classical monocytes, non-classical monocytes, and intermediate monocytes between CN (CN−: CN −ve), pre-clinical (CN+: CN +ve), and AD with dementia (Dementia: MCI +ve and AD) individuals. Bar graphs illustrated the boxplot distribution of individual measurements with “x” denoting the mean. Three-group comparison was determined by one-way ANOVA followed by multiple comparison using Dunnett’s post-hoc test (solid line). (**E**–**J**) Associations between monocyte P2X7 expressions, PACC, and CSF biomarker. Correlation r and *p* values were calculated by Pearson product-moment correlational analysis. The “grey” band indicated the 95% confidence interval of the black linear regression line. ns: no significance.

We drew at random 88 participants from the Australian Imaging, Biomarker & Lifestyle Flagship Study of Ageing (AIBL) study (Table 1) and we used APC-conjugated anti-P2X7 antibodies to study the surface expression of P2X7 on peripheral leukocytes. The main results of these comparisons were shown in Figure 1. Monocyte surface expression of P2X7 was reduced significantly in Aβ +ve cases compared with Aβ −ve controls (Figure 1A). Regarding subpopulations of monocytes, P2X7 expressions on CD14^+^CD16^−^ classical monocytes, CD14^dim^CD16^+^ non-classical monocytes, and CD14^+^CD16^+^ intermediate monocytes were all reduced in Aβ +ve cases compared with Aβ −ve controls with effect sizes of −0.487, −0.503, and −0.514, respectively (Figure 1A; Table S1). Also, P2X7 surface expression on non-classical monocytes was the lowest while that on intermediate monocytes was the highest among monocyte subtypes (Figure 1A). This cohort was further categorised into CN −ve, CN +ve, and dementia (MCI +ve and AD) groups. P2X7 expressions on monocyte subpopulations were significantly different between the three groups (Figure 1B–D), in which P2X7 expression on classic monocytes in CN +ve group and dementia group had effect sizes of −0.564 and −0.672, respectively (Table S2). While the distribution of P2X7 expression was spreading in three groups, more CN −ve individuals presented high P2X7 level, while more CN +ve and dementia individuals presented low P2X7 level (Figure 1B–D). Moreover, P2X7 expression on classical monocytes trended towards a positive association with PACC, while P2X7 expressions on other monocyte subpopulations failed to be associated with PACC (Figure 1E–G). Regarding CSF biomarkers, P2X7 expression on non-classical monocytes was associated negatively with Aβ_1-42_/T-tau ratio in CSF, while P2X7 expressions on other monocyte subpopulations trended towards negative associations with this CSF biomarker (Figure 1H–J).

**Figure 2:**
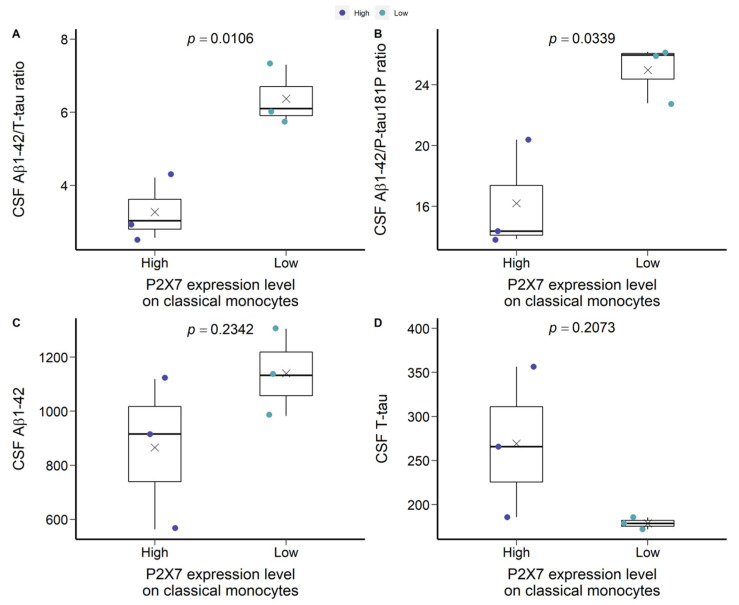
Characteristics of P2X7-low CN −ve and P2X7-high CN −ve groups in the discovery cohort. (**A**) CSF Aβ_1-42_/T-tau ratio. (**B**) CSF Aβ_1-42_/P-tau181P ratio. (**C**) CSF Aβ_1-42_ concentration (μg/L). (**D**) CSF T-tau concentration (μg/L). Two group comparison was determined by *t*-test.

Of note, in CN −ve group, a bimodal distribution of P2X7 expression was observed, which was separated by the mean of mean fluorescence intensity (MFI) of P2X7 on classical monocytes (Figure 1B). The group below the mean demonstrated similar P2X7 expressions compared to CN +ve and dementia individuals. Therefore, we arbitrarily separated CN −ve group into two populations, defining the cohort over the mean as P2X7-high CN −ve and the cohort below the mean as P2X7-low CN −ve (Figure 1B). We compared Aβ burden, brain volumetrics, and neuropsychological assessments to characterise the P2X7-low CN −ve group. P2X7-low CN −ve individuals presented significantly higher Aβ_1-42_/T-tau and Aβ_1-42_/P-tau181P ratios in CSF compared with P2X7-high CN +ve individuals (Figure 2A,B). Moreover, these individuals were likely to present higher Aβ burden and lower T-tau burden in CSF (Figure 2C,D).

**Figure 3:**
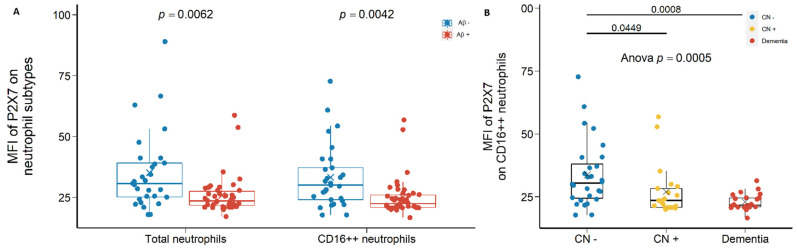
P2X7 expressions on neutrophils in the discovery cohort. (**A**) P2X7 expressions on total neutrophils (*left*) and CD14^−^CD16^++^ neutrophils (*right*) between in Aβ −ve controls (Aβ−) and Aβ +ve cases (Aβ+). Bar graphs illustrated the boxplot distribution of individual measurements with “x” denoting the mean. Two group comparison was determined by *t*-test. (**B**) P2X7 expressions on CD14^−^CD16^++^ neutrophils between CN (CN−: CN −ve), pre-clinical (CN+: CN +ve), and AD with dementia (Dementia: MCI +ve and AD) individuals. Bar graphs illustrated the boxplot distribution of individual measurements with “x” denoting the mean. Three-group comparison was determined by one-way ANOVA followed by multiple comparison using Dunnett’s post-hoc test (solid line).

Second, P2X7 expressions on total neutrophils and CD16^++^ neutrophils were also reduced significantly in Aβ +ve cases compared with Aβ −ve controls with effect sizes of −0.581 and −0.625, respectively (Figure 3A; Table S1). Most importantly, P2X7 on CD16^++^ neutrophils were significantly lower in CN +ve group and dementia group compared with CN −ve group, with effect sizes of −1.171 and −1.067, respectively (Figure 3B; Table S2).

**Figure 4:**
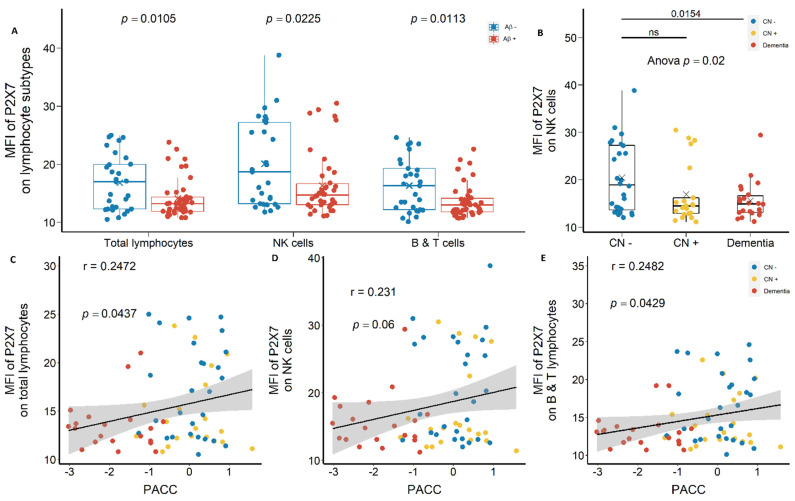
P2X7 expressions on lymphocytes in the discovery cohort. (**A**) P2X7 expressions on total lymphocytes (*left*), CD14^−^CD16^+^ NK cells (*middle*), and CD14^−^CD16^−^ B and T lymphocytes (*right*) between Aβ −ve (Aβ−) and Aβ +ve (Aβ+). Bar graphs illustrated the boxplot distribution of individual measurements with “x” denoting the mean. Two group comparison was determined by *t*-test. (**B**) P2X7 expressions on NK cells between CN (CN−: CN −ve), pre-clinical (CN+: CN +ve), and AD with dementia (Dementia: MCI +ve and AD) individuals. Bar graphs illustrated the boxplot distribution of individual measurements with “x” denoting the mean. Three-group comparison was determined by one-way ANOVA followed by multiple comparison using Dunnett’s post-hoc test (solid line). (**C**–**E**) The associations between P2X7 expressions and PACC. Correlation r and *p* values were calculated by Pearson product-moment correlational analysis. ns: no significance.

Lymphocyte surface expression of P2X7 also demonstrated similar results. P2X7 expression on CD14^−^ total lymphocytes, CD14^−^CD16^+^ NK cells, and CD14^−^CD16^−^ T and B lymphocytes were reduced significantly in Aβ +ve cases compared with Aβ −ve controls with effect sizes of −0.572, −0.509, and −0.559, respectively (Figure 4A; Table S1). P2X7 expression on NK cells were the highest among lymphocyte subtypes (Figure 4A), which was significantly lower in CN +ve and dementia groups compared with CN −ve group, with effect sizes of −0.550 and −0.758, respectively (Figure 4B; Table S2). Moreover, P2X7 expressions on all lymphocyte subtypes were associated positively with neuropsychological score (PACC) (Figure 4C–E).

#### 2.1.2. Reduced Monocyte Surface P2X7 Expressions in Aβ +Ve Cases in Validation Cohort

**Figure 5:**
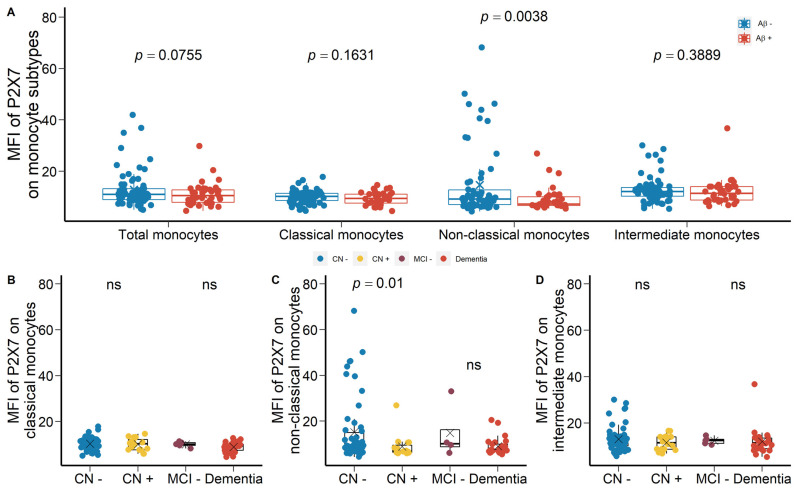
P2X7 expressions on monocytes in the validation cohort A. (**A**) P2X7 expressions on total monocytes (*left 1*), CD14^+^CD16^−^ classical monocytes (*left 2*), CD14^dim^CD16^+^ non-classical monocytes (*right 2*), and CD14^+^CD16^+^ intermediate monocytes (*right 1*) between Aβ −ve controls (Aβ−) and Aβ +ve cases (Aβ+). Two group comparison was determined by *t*-test. (**B**–**D**) Comparisons of P2X7 expressions on classic monocytes, non-classical monocytes, and intermediate monocytes between CN (CN−: CN −ve) and pre-clinical (CN+: CN +ve) individuals. Comparisons of P2X7 expressions on classic monocytes, non-classical monocytes, and intermediate monocytes between prodromal (MCI−: MCI −ve) and AD with dementia (Dementia: MCI +ve and AD) individuals. Two group comparison was determined by *t*-test. ns: no significance.

After identifying the global downregulation of leukocyte surface P2X7 in Aβ +ve cases in the discovery cohort, we drew at random a second group of 111 participants from the AIBL study to validate our results (the validation cohort A, Table 1). In validation cohort A, FITC-conjugated anti-P2X7 antibodies were used to study P2X7, so the value of MFI was not comparable between the two cohorts. A global downregulation of cell surface P2X7 expressions on all leukocyte subtypes were observed (Table S3). Both monocyte and neutrophil surface P2X7 expressions showed significant differences between Aβ +ve cases and Aβ −ve controls (Table S3). P2X7 expression on non-classical monocytes were significantly lower in Aβ +ve cases compared with Aβ −ve controls with effect size of −1.233 (Figure 5A; Table S3). The validation cohort were further categorised into CN −ve, CN +ve, MCI −ve, and dementia groups. By comparing CN −ve and CN +ve groups, P2X7 expression on non-classical monocytes was significantly lower in CN +ve group with effect size of −1.183 (Figure 5C; Table S4). By comparing MCI −ve and dementia groups, no differences were observed (Figure 5B–D).

**Figure 6:**
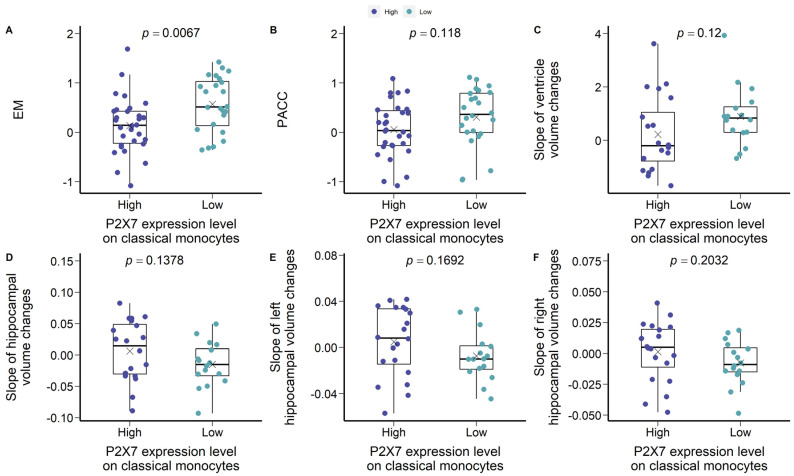
Characteristics of P2X7-low CN −ve and P2X7-high CN −ve groups in the validation cohort A. (**A**) EM. (**B**) PACC. (**C**) Slope of ventricle volume changes per year. (**D**) Slope of hippocampal volume changes per year. (**E**) Slope of left hippocampal volume changes per year. (**F**) Slope of right hippocampal volume changes per year. Two group comparison was determined by *t*-test.

To repeat the analysis of the bimodal cohorts at the discovery stage, we separated the P2X7-high CN −ve group from the P2X7-low CN −ve group by the mean MFI of P2X7 on classical monocytes (Figure 5B). P2X7-low CN −ve individuals were characterised by higher EM and PACC compared with P2X7-high CN −ve individuals (Figure 6A,B). Regarding brain volumetrics, P2X7-low CN −ve individuals were likely to present a faster expanding rate of ventricle and faster shrinking rate of hippocampus (Figure 6D–F).

### 2.2. Strong Correlation between P2X7 Expressions and Integrin Expressions

**Figure 7:**
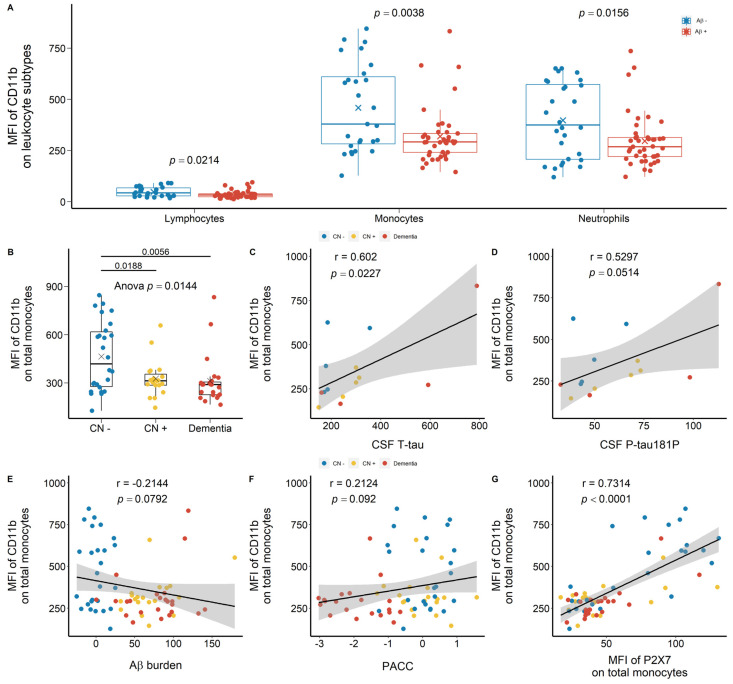
CD11b expressions on peripheral leukocytes in the discovery cohort. (**A**) CD11b expressions on total lymphocytes (*left*), total monocytes (*middle*), and total neutrophils (*right*) between Aβ −ve (Aβ−) and Aβ +ve (Aβ+). Bar graphs illustrated the boxplot distribution of individual measurements with “x” denoting the mean. Two group comparison was determined by *t*-test. (**B**) CD11b expressions on total monocytes between CN (CN−: CN −ve), pre-clinical (CN+: CN +ve), and AD with dementia (Dementia: MCI +ve and AD) individuals. Bar graphs illustrated the boxplot distribution of individual measurements with “x” denoting the mean. Three-group comparison was determined by one-way ANOVA followed by multiple comparison using Dunnett’s post-hoc test (solid line). (**C**–**F**) Associations between monocytic CD11b expressions, CSF biomarkers (μg/L), Aβ burden (CL) measured by PET, and PACC. (**G**) Association between leukocyte CD11b expressions and P2X7 expressions. Correlation r and *p* values were calculated by Pearson product-moment correlational analysis. The “grey” band indicated the 95% confidence interval of the black linear regression line.

Regarding integrins CD11b and CD11c, similar trends were observed in the discovery cohort. CD11b was highly expressed on monocytes and neutrophils compared with lymphocytes (Figure 7A). In Aβ +ve cases, lower CD11b expressions on monocytes and neutrophils were observed with effect sizes of −0.651 and −0.526, respectively (Figure 7A; Table S1). By categorising the discovery cohort into CN −ve, CN +ve, and dementia groups, both CN +ve and dementia individuals presented significantly lower CD11b expression on monocytes with effect sizes of −1.311 and −0.861, respectively (Figure 7B; Table S1). CD11b expression on monocytes were further associated positively with T-tau and P-tau181P concentrations in CSF and PACC (Figure 7C,D,F). Monocytic CD11b expression further trended towards a negative association with Aβ burden in the brain (Figure 7E). Additionally, monocyte surface CD11b expression was associated strongly, positively with monocyte surface P2X7 expression (Figure 7G). Regarding CD11b expression on peripheral neutrophils, we did not observe significant differences across CN −ve, CD +ve, and dementia groups (Figure S1A). Neutrophil surface CD11b expression was associated positively with T-tau concentration in CSF and neutrophil surface P2X7 expression (Figure S1B,F), but no significant associations were found with CSF P-tau181P, Aβ burden, and PACC (Figure S1C–E).

**Figure 8:**
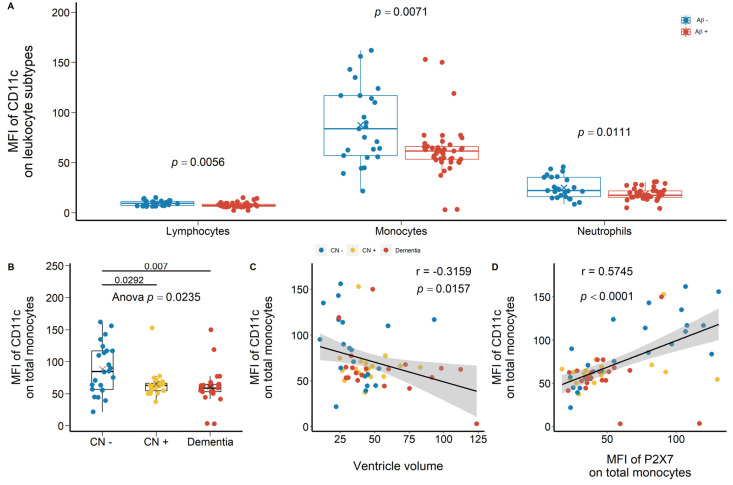
CD11c expressions on peripheral leukocytes in the discovery cohort. (**A**) CD11c expressions on total lymphocytes (*left*), total monocytes (*middle*), and total neutrophils (*right*) between Aβ −ve controls (Aβ−) and Aβ +ve cases (Aβ+). Bar graphs illustrated the boxplot distribution of individual measurements with “x” denoting the mean. Two group comparison was determined by *t*-test. (**B**) CD11c expressions on total monocytes between CN (CN−: CN −ve), pre-clinical (CN+: CN +ve), and AD with dementia individuals (Dementia: MCI +ve and AD). Bar graphs illustrated the boxplot distribution of individual measurements with “x” denoting the mean. Three-group comparison was determined by one-way ANOVA followed by multiple comparison using Dunnett’s post-hoc test (solid line). (**C**) The association between monocyte CD11c expression and ventricle volume. (**D**) The association between monocyte CD11c expression and P2X7 expression. Correlation r and *p* values were calculated by Pearson product-moment correlational analysis. The “grey” band indicated the 95% confidence interval of the black linear regression line.

Another integrin of interest was CD11c, which was highly expressed on monocytes compared with lymphocytes and neutrophils (Figure 8A). CD11c expressions on lymphocytes, monocytes, and neutrophils were reduced significantly in Aβ +ve cases compared with Aβ −ve controls with effect sizes of −0.701, −0.639, and −0.580, respectively (Figure 8A; Table S1). Compared with CN −ve individuals, monocyte surface CD11c expression in CN +ve and dementia individuals was significantly lower with effect sizes of −1.054 and −1.249 (Figure 6B; Table S1). Moreover, CD11c expression on monocytes was associated negatively with ventricle volume while associated positively with P2X7 expression on monocytes (Figure 8C,D). Similarly, neutrophil surface CD11c expression was significantly lower in CN +ve and dementia individuals compared with CN −ve individuals (Figure S2A). It was strongly associated with neutrophil surface P2X7 expression, while trended towards a significant association with ventricle volume (Figure S2B,C).

### 2.3. Altered Percentage of Monocyte Subtypes in MCI and AD

We used traditional markers, CD14 and CD16, to gate for CD14^+^CD16^−^ classic monocytes, CD14^dim^CD16^+^ non-classic monocytes, and CD14^+^CD16^+^ intermediate monocytes, followed by calculating their relative percentages in total monocyte population. Although the percentages of these monocyte sub-populations did not differ significantly between Aβ +ve cases and Aβ −ve controls, they were associated with CSF measurements of Aβ_1-42_, T-tau, and P-tau181P (Figure S3). Non-classical monocytes and intermediate monocytes were associated negatively, strongly with both CSF T-tau and P-tau181P concentrations (Figure S3B,C,E,F). Regarding CSF ratios, intermediate monocytes and non-classical monocytes were associated strongly, positively with Aβ_1-42_/T-tau and Aβ_1-42_/P-tau181P ratios in CSF (Figure S3H,J–L), while classical monocytes were associated negatively with these ratios (Figure S3G,J). In validation cohort A, no significant correlations were observed.

We also used anti-HLA-DR antibodies to identify HLA-DR^+^ monocyte subpopulation. In the discovery cohort, the percentage of HLA-DR^+^ monocytes in total monocyte population was lower in Aβ +ve cases compared with Aβ −ve controls, supported by higher HLA-DR^−^ monocytes in Aβ +ve cases (Figure S4A). By categorising the discovery cohort into CN −ve, CN +ve, and dementia, HLA-DR^+^ monocytes were significantly lower in dementia group compared with CN −ve controls (Figure S4B). Furthermore, HLA-DR^+^ monocytes were associated negatively with ventricle volume and its rate of change (Figure S4C,D), while associated positively with the rate of change of cortical grey matter volume (Figure S4E). Regarding neuropsychological assessments, HLA-DR^+^ monocytes were associated positively with PACC (Figure S4F). HLA-DR^+^ monocytes were further associated with P2X7 expressions on total monocytes and classical monocytes (Figure S4G,H). To validate these findings, we further drew another independent cohort comprising of 88 participants from the AIBL study (Validation cohort B, Table 1). We failed to observe significant differences of HLA-DR^+^ monocytes between Aβ +ve cases and Aβ −ve controls (S5A). By categorising the validation cohort B into CN −ve, CN +ve, MCI− ve, and dementia groups, the percentage of HLA-DR^−^ monocytes were significantly higher in dementia individuals compared with MCI −ve individuals (Figure S5B). Additionally, HLA-DR^+^ monocytes were associated positively with the rate of change of cortical white matter volume (Figure S5C). Regarding neuropsychological scores, HLA-DR+ monocytes were associated positively with EM and its rate of change (Figure S5D,E), while HLA-DR^−^ monocytes was associated negatively with EM and its rate of change (Figure S5F,G).

### 2.4. Unchanged Polygenic Risk Scores (PRS) of P2X7 in AD

We developed a PRS comprised of 12 *P2RX7* and *P2RX4* single nucleotide polymorphisms (SNP), which were associated with neurodegeneration and P2X7 function. A total of 12 neurodegeneration-related SNPs were selected from our previous functional studies and literature, including 12 SNPs in *P2RX7* and one SNP in *P2RX4* (Table S5). Among the 12 SNPs, ten of them were associated with the pore formation function of P2X7, while three of them were associated with the innate phagocytic function of P2X7 (Table S5). Traditionally, the effect sizes of risk alleles are determined by summary statistics of relevant genomic studies. In this study, we arbitrarily determined their effect sizes according to the functional assessments of *P2RX7* SNPs by our group and literature (Table S5). Given the dual functionality of P2X7, two PRS were generated. PRS-pore was determined by ten genetic variants associated with pore formation function of P2X7 (Table S5). PRS-phago was then determined by three genetic variants associated with the innate phagocytic function of P2X7 (Table S5). A total of 1738 participants were recruited from the AIBL database (*n* = 919) and the Alzheimer’s Disease Neuroimaging Initiative (ADNI) database (*n* = 819). By excluding samples with missing demographics or genotyping data, 900 AIBL participants and 786 ADNI participants remained for PRS analysis. The characteristics of *P2RX7* and *P2RX4* SNPs in the AIBL and the ADNI cohort were summarised in Table 2. We first used PLINK 1.9 to study whether *P2RX7* and *P2RX4* SNPs were associated with the clinical diagnosis. The only SNP that reached statistical significance was rs17525809 (V76A) in the AIBL cohort (Table S6; *p* = 0.05). Subsequently, we calculated two PRS depending on the associated functions of these SNPs. The summary of PRS-pore and PRS-phago in each cohort and the combined cohort were presented in Table 2, no differences were observed between cases and controls.

## 3. Discussion

### 3.1. Reduced Peripheral Leukocyte Surface P2X7 Expressions in Aβ +Ve Cases

Dysregulated immune responses have been raised as a possible contributor to AD pathogenesis and this study compared P2X7 expressions on adaptive and innate immune-related cells between Aβ +ve cases and Aβ −ve controls. We demonstrated globally reduced P2X7 expressions on peripheral leukocytes in Aβ +ve cases, including lymphocytes, monocytes, and neutrophils, as previously reported by our group [11]. Leukocyte surface P2X7 expressions were further associated with brain atrophy, neuropsychological estimates, and CSF biomarkers. These results indicated that patients with low leukocyte P2X7 expressions were likely to present higher Aβ burden, more severe brain atrophy, more compromised cognitive, learning, and memorising abilities in AD. While our results demonstrated P2X7 downregulation on monocytes, P2X7 upregulation was found in microglia near Aβ plaques in the brains of AD patients and AD mice models [16]. P2X7 is a dual-functional purinergic receptor that acts as an ATP-induced pro-inflammatory ion channel in ATP-rich environment [13]. Its pro-inflammatory function has been well associated with AD [13]. One possible mechanism of P2X7 upregulation in AD brain is due to the overexpressed transcriptional factor specificity protein 1 (SP1), as shown in mice model of AD [17]. Recent genomic study of AD has revealed that SP1 is one of the over-represented motifs found in the active enhancers of myeloid cells and these enhancers might upregulate many AD GWAS risk genes [18]. P2X7 upregulation in the central pool of AD patients and in mice models of AD has been widely demonstrated, but the reasons underlining P2X7 downregulation in the periphery awaits further elucidation.

### 3.2. Are Low P2X7, CD11b, and CD11c Expressions Indicative of Pre-Clinical AD?

Following recognising differential P2X7 expressions in individuals with different PET-Aβ status, we further compared P2X7 expressions by including both PET-Aβ status and clinical symptoms. In the discovery cohort, due to the lack of prodromal AD patients (MCI −ve), only three groups were categorised: CN individuals (CN −ve), pre-clinical AD patients (CN +ve), and AD patients with dementia (Dementia: MCI +ve and AD). P2X7 expressions on monocytes and neutrophils were significantly lower in pre-clinical AD patients compared with CN individuals. P2X7 downregulation may happen early at the pre-clinical stage of AD and stayed stable throughout disease course, but the underlying mechanism remains unknown. Interestingly, P2X7 expressions on lymphocytes and monocytes resembled bimodal distribution, in which over half of CN individuals expressed high level of P2X7, while the others expressed low level of P2X7. Compared with CN individuals with high level of P2X7, CN individuals with low level of P2X7 were characterised by higher CSF Aβ_1-42_/T-tau ratio, higher CSF Aβ_1-42_/P-tau181P ratio, and better cognition (higher EM). They were likely to present faster expanding rate of ventricle and faster shrinking rate of hippocampus. AD progression is characterised by low CSF Aβ_1-42_, high CSF Tau, high CSF pTau181, low CSF Aβ_1-42_/Tau ratios [19], and low EM. Therefore, CN individuals with low level of P2X7 were not expected to present high CSF Aβ_1-42_/T-tau ratio or high EM. It suggested that CN individuals with low P2X7 level may undergo uncharacterised pathological changes and progress into the pre-clinical stage of AD soon, but more investigations are required to elucidate P2X7 downregulation in AD pathogenesis and progress. This pre-clinical P2X7 downregulation may be associated with its function as a scavenger receptor responsible for innate phagocytosis in serum-free environment [13]. In the central pool of AD individuals, ATP-induced P2X7 pore activation dissociates transmembrane P2X7 from intracellular actin cytoskeleton and attenuates microglial phagocytic capacity [20]. However, the required concentration of ATP for P2X7 pore activation can be rarely achieved in bloodstream or CSF (>100 µM, but usually 1–5 mM depending on ambient divalent cations) [13]. This suggests that a higher proportion of P2X7 receptors may tightly attach to actin cytoskeleton, directing more P2X7 towards scavenger function mediating phagocytosis [11]. Whether peripheral P2X7 downregulation at the pre-clinical stage of AD is associated with compromised phagocytotic ability or attenuated pro-inflammatory responses awaits further investigation.

Integrin downregulation in Aβ +ve cases was also observed, particularly on professional phagocytes—monocytes and neutrophils. CD11b and CD11c play important roles in the recruitment and migration of mononuclear phagocytes, cell-cell contact formation, and immune cell signalling [21]. They also pair with the β_2_ integrin subunit, CD18, to form complement receptor 3 (CR3) and 4 (CR4) respectively, mediating complement-coated particle phagocytosis [21]. Several studies had identified increased CD11b and CD11c levels in the brains of AD patients compared with CN individuals [22], but our results reported the contradictory facts in bloodstream. Both integrins were downregulated significantly at the pre-clinical stage of AD. Leukocyte surface integrin expressions were further associated with CSF biomarkers, cognitive decline (PACC), and ventricle enlargement. Interestingly, the simultaneous downregulation of integrins and P2X7 was demonstrated, indicating that the pre-clinical AD patients expressed both low levels of P2X7 and integrins on their monocytes and neutrophils, which stayed low throughout disease course. Their downregulation in the periphery might significantly compromise the phagocytic and migrating abilities of monocytes and neutrophils from the pre-clinical AD stage to the AD with dementia stage.

### 3.3. Altered Peripheral Monocyte Subpopulations in AD

Given that our discoveries of P2X7 and integrins converge on innate immunity and monocytes, we further dissected monocyte population into CD14^+^CD16^−^ classical monocytes, CD14^dim^CD16^+^ non-classical monocytes, and CD14^+^CD16^+^ intermediate monocytes to study their associations with AD. Progression of AD can be characterised by low CSF Aβ_1-42_, high CSF T-tau, high CSF P-tau181P, and low CSF Aβ_1-42_/T-tau ratio [19]. As AD progressed, we observed fewer intermediate monocytes, fewer non-classical monocytes, but more classical monocytes in bloodstream in the discovery cohort. Classical monocytes are professional phagocytes, occupying 80% of total population [23]. Intermediate monocytes are expertise at antigen presentation, cytokine secretion, apoptosis regulation, and transendothelial migration [23]. The infiltration of intermediate monocytes into the CNS due to leaky BBB remains debatable and our findings provide indirect evidence of infiltrative intermediate monocytes into the brain [24]. Furthermore, we also studied HLA-DR^+^ monocytes to investigate adaptive immunity in AD progression. Our discovery cohort illustrated significantly fewer HLA-DR^+^ monocytes in AD cases with dementia compared with CN individuals, as supported by more HLA-DR^−^ monocytes in the AD cases with dementia. Previous study showed reduced HLA-DR^+^CD14^+^ monocytes and increased HLA-DR^+^CD16^+^ monocytes during AD progression. HLA-DR^+^ monocytes were further associated with brain atrophy, neuropsychological estimates, and leukocyte surface P2X7 expressions. These observations further consolidate the involvement of both innate and adaptive immunity in AD, suggesting compromised phagocytic capacity at the pre-clinical stage of AD and altered antigen presentation ability of professional mononuclear phagocytes at the dementia stage of AD. Moreover, reduced CD16^−^ monocytes (classical) and increased CD16^+^ monocytes (non-classical and intermediate) in the blood circulation of AD patients were also noted and whether this phenomenon is associated with infiltrative monocytes into the CNS awaits further investigation.

### 3.4. P2X7 Alterations in AD Were Not Caused by Genetic Factors

To further assess the association between P2X7 function and AD, we selected 12 SNPs in *P2RX7* and *P2RX4* genes that are known to inhibit or promote the pore formation function or phagocytic function of P2X7. However, no significant association was found between the *P2RX7* gene and AD. This observation is reasonable because no *P2RX7* SNP has been identified as a risk allele in AD GWAS [4]. Further PRS analysis with added functional notes did not show any difference between MCI/AD cases and CN controls either. Therefore, the changes of P2X7 expression on leukocytes may not be determined by genetic factors but may be regulated by environmental factors instead. It suggested that P2X7 expression level is highly modifiable as AD progressed, making P2X7 a promising therapeutic target.

### 3.5. Limitations of Current Study

All participants were recruited in Perth and Melbourne through the AIBL study, and the ethnic background was limited to 99.9% Caucasians who were fluent in English. The application of our findings to general population would require more investigations by involving participants with various ethnic backgrounds. Second, our study showed interesting associations with CSF measurements of Aβ_1-42_, Tau, and pTau181. However, CSF data were scarce, and we need to collect more CSF measurements to validate our findings.

## 4. Materials and Methods

### 4.1. Flow Study: Study Individuals, Ethical Approvals, and Blood Samples

A total of 287 participants were drawn at random from the AIBL study. The AIBL study is a dual-site, longitudinal, prospective, observational study that integrated data from neuroimaging, biomarkers, lifestyle, clinical, and neuropsychological analyses [25]. This is a two-stage study, comprising of a discovery stage (*n* = 88), a validation stage A for the P2X7 study (*n* = 111), and a validation stage B for the HLA-DR study (*n* = 88) (Table 1). The participants in all stages were independent and all accident duplicated participants had been removed. All participants were over 65 years old and fluent in English. The clinical classification of disease status, namely CN, MCI, and AD dementia, was assessed by neuropsychological examinations, as defined by NINCDS-ADRDA criteria [26,27,28]. The summary of participants’ demographics and clinical characteristics was demonstrated in Table 1.

Peripheral whole blood was collected via venepuncture between 8:00 a.m. and 10:30 a.m. following overnight fasting. Whole blood was kept in EDTA anti-coagulant Vacutainer^®^ tube (Becton Dickinson Biosciences, Franklin Lakes, NJ, USA) and was kept on ice during transportation. Processing of whole blood was completed within three hours after collection.

This study was approved by the Eastern Health Research and Ethics Committee (Ref: E05/1011, since Sep 2010) and conducted according to Declaration of Helsinki principles. All participants and patient caregivers completed written informed consent. All clinical and demographic information was masked until the collection of all laboratory measurements.

### 4.2. PRS Study: Study Individuals and Ethical Approvals

A total of 1738 participants were recruited from the AIBL database (*n* = 919) and the ADNI database (*n* = 819). ADNI was initiated in 2003 in the United States and Canada, aiming at the development of standardized imaging procedure and biomarker assessment in normal, preclinical, and prodromal patients with AD. The demographics and characteristics of these two datasets were summarised in Table 2. Regional ethical committees of all institutions included in ADNI approved of the study and all subjects have provided informed consent.

### 4.3. Flow Study: Materials and Immune Staining

Fluorophores conjugated antibodies were purchased from BD Bioscience (Franklin Lakes, NJ, USA) and DAKO (Agilent Technologies, Santa Clara, CA, USA). The anti-human P2X7 monoclonal antibodies were produced from the L4 clone in house [29] and conjugated with Alexa 647 or Alexa 488 using the antibody conjugation kit from Molecule Probes (Thermo Fisher Scientific, Waltham, MA, USA).

Cell surface staining was carried out following the BD standard protocol. Aliquots of 100 µL of fresh whole blood were added into fluorescence-activated cell sorting (FACS^®^) tubes with pre-mixed antibody cocktails. An autofluorescence tube containing only whole blood and an IgG isotype control (BD Australia, Macquarie Park, NSW, Australia) tube were prepared for each AIBL sample. Titration of each antibody was determined by saturation tests. Blood/antibody mixture was incubated for 15 min at room temperature with gentle shake, followed by incubating with 2 mL of BD FACS Lysing solution (Cat#555899) for another 15 min. 2 mL of PBS was added to each FACS^®^ tubes, followed by centrifuging at 1400 rpm for 3 min. Supernatant was discarded and leukocytes were resuspended into 200 uL of PBS. Leukocytes were then analysed using FACSCalibur^TM^ (BD Biosciences) and flow results were primarily analysed using FlowJo software (V10, FlowJo, LLC, Ashland, OR, USA).

### 4.4. Flow Study: Magnetic Resonance Imaging (MRI)

The MRI scans of every participant were performed as previously described [30,31]. Images were acquired using a standard three-dimensional magnetisation-prepared rapid gradient echo sequence at 3 T, with in-plane resolution 1 × 1 mm, slice thickness 1.2 mm, repetition time (TR)/echo time (TE)/T1 = 2300/2.98/900, flip angle 9°, field of view 240 × 256, and 160 slices. Axial T2-weighted MR images were acquired using a standard two-dimensional turbo spin echo sequence at 3 T, with in-plane resolution 0.9375 × 0.9375 mm, slice thickness 3 mm, TR/TE = 3400/101, flip angle 150°, field of view 228 × 256, and 48 slices. All T1-weighted (T1W) images were first corrected for bias field using the N4 algorithm [32] and smoothed using anisotropic filtering. T2W images were motion corrected using inverse interpolation [33]. For each participant, all images were first segmented into grey matter (GM), white matter (WM), ventricle, hippocampus, and cerebrospinal fluid (CSF) in their native space using an in-house implementation of the Expectation Maximization Segmentation algorithm [32].

### 4.5. Flow Study: PET-Aβ Imaging

Most participants underwent PET-Aβ imaging as previously described [34]. All PET-Aβ scans were spatially normalised using CapAIBL [31] and quantified using the Centiloid (CL) scale [35,36]. CL of 25 was used as the cut-off point of PET-Aβ positivity.

### 4.6. Flow Study: EM Score

The rationale, development, and validation for the EM composite scores had been previously detailed [37,38]. Firstly, we standardised scores for the Scores for the California Verbal Learning Test (Second Edition, CVLT-II) delayed recall, Logical Memory delayed recall, and Rey Complex Figure Test delayed recall, using the baseline mean and the baseline mean and standard deviation for the entire sample of the CN older adult group in AIBL. The EM composite score was then formed by averaging the standardised scores.

### 4.7. Flow Study: PACC Score

The rationale, development, and validation for the PACC had been previously described [39,40]. We first standardised scores for the California Verbal Learning Test (Second Edition, CVLT-II) delayed recall, Logical Memory delayed recall, Digit Symbol Coding, and MMSE, using the baseline mean and standard deviation for the entire sample of the cognitively normal older adult group in AIBL. The PACC was then calculated by averaging the standardised scores.

### 4.8. Flow Study: CSF Measurements of Biomarkers

The collection, processing, measurement, and long-term storage of CSF samples had been previously described [6]. CSF was collected in the morning by routine lumbar puncture after overnight fasting, using a similar protocol recommended by the Alzheimer’s Biomarkers Standardisation Initiative [41]. CSF was tested routinely using the INNOTEST^®^ kit assay (Innogenetics, now Fujirebio Europe N.V., Ghent, Belgium) for Aβ_1-42_, total Tau, and pTau181.

### 4.9. Flow Study: Calculation of Progressive Changes

As a longitudinal study, most participants of AIBL have undergone long-term follow-up concerning brain imaging, cognitive assessments, and other pathological examinations in 18-month intervals. Most participants in this study had 2–6 visits to AIBL clinics, which enabled researchers to investigate progressive changes of their pathological tests. The yearly slopes of PET-Aβ scans, MRI, EM, and PACC were calculated from at least two time points using the Excel “Slope” function.

### 4.10. Flow Study: Statistical Rational

Before conducting statistical analysis, qqPlot() and barlett.test() in R were used to assess normality and homogeneity of variances, respectively. Sample characteristics of age, sex, APOE ε4 carriers, year of education, and neuropsychological assessments were compared using one-way ANOVA (continuous variables) and chi-square test (categoric variables). The comparisons of leukocyte receptors were first performed between Aβ +ve cases and Aβ −ve controls by *t*-test using the oneway.test() function in R. In discovery cohort (no prodromal cases), the subsequent comparisons of receptors between CN −ve, CN +ve, and demented cases were determined by one-way ANOVA using oneway.test() function in R. Its post-hoc tests were calculated by Dunnett’s test using the DunnettTest() in R. In validation cohort, the comparisons between CN +ve and CN −ve and the comparisons between demented AD cases and prodromal AD cases were calculated by *t*-test using the oneway.test() function in R. The correlations between receptors and Aβ burden, brain volumes, neuropsychological assessments, and CSF measurements of Aβ_1-42_, T-tau, and P-taau181P were determined by calculating Pearson product-moment correlation coefficients (r) using the cor() function in R. After dissecting the CN controls by mean P2X7 MFI, two-group comparisons were determined by student t-test using oneway.test() in R. All statistical plots were generated using the ggplot() function in R. R version 3.6.3 (29 February 2020)—“Holding the Windsock” Copyright (C) 2020 The R Foundation for Statistical Computing Platform: x86_64-w64-mingw32/x64 (64-bit).

### 4.11. PRS Study: Genotype Data, PRS Calculation, and Data Analysis

Data was available across 919 individuals in AIBL database and 809 individuals in ADNI database. Prior to data analysis, data from each databased were QC’ed separately using PLINK (1.9, Shaun Purcell, https://pngu.mgh.harvard.edu/purcell/plink/ accessed on 12 October 2013). A total of 12 neurodegeneration-related *P2RX7* and *P2RX4* SNPs were selected from our previous functional studies and literature, including 11 SNPs in *P2RX7* and one SNP in *P2RX4* (Table 2; Tables S6 and S7). The classical PRS is an estimate of an individual’s genetic liability to a disease by their genotype profile and effect sizes of each allele determined by genome-wide association study [42]. Instead of classic procedure, the effect sizes of risk alleles in this study were determined by functional assessments of *P2RX7* SNPs by our group and literature (Table S5). Two PRS were calculated. The first PRS-pore was determined by 10 genetic variants that had been associated with pore formation of P2X7 (Table S5). The second PRS-phago was determined by three genetic variants that had been associated with the innate phagocytic function of P2X7 (Table S5).

## 5. Conclusions

We identified the downregulation of P2X7, CD11b, and CD11c on peripheral leukocyte surface at the pre-clinical stage of AD and they were further associated with brain atrophy, cognition decline, and CSF biomarkers of AD. Their significant associations with the current diagnostic standards of AD strengthened their involvement in early stage of disease course, suggesting that the dysfunction of pro-inflammatory responses, phagocytic functions, and migrating abilities of circulating phagocytes may happen at the pre-clinical stage of AD and stay compromised throughout disease course. On the contrary, the antigen presentation function of circulating monocytes may be altered at the dementia stage of AD. Our results consolidate that AD is a systemic disease modulated by both central and peripheral immune responses, in which altered innate immune responses may happen at asymptomatic stage of AD while altered adaptive immune responses happen at symptomatic stage of AD. Most interestingly, low level of P2X7 expression on monocytes might indicate patients who had faster shrinking rate of hippocampus but normal Aβ burden and cognitive function. Given that altered immune responses had been raised as a strong contributor to AD progression, as supported by many genetic studies, our study deepens the understanding of the peripheral immune dysfunction at the pre-clinical stage of AD. This not only improves the understanding of the role of immunity in AD pathogenesis, but also provides novel insights into biomarker discovery. Leukocyte surface expression of immune-related receptors, such as P2X7 and integrins, might be promising biomarkers of AD, which might facilitate the diagnosis and prognosis of pre-clinical AD patients. Given the over 60 microglial-specific AD risk genes identified by AD GWAS, it is promising to study these immune-related markers on professional phagocytes to improve our understanding of immune involvement in AD and to provide more insights into biomarker discovery and therapeutic development.

## Figures and Tables

**Table 1:** Demographic characteristics of our participants.

Demographics	Discovery		*p* ^1^	Validation A ^2^		*p*	Validation B ^3^		*p*
	Control (≤25 CL)	Case (>25 CL)		Control (≤25 CL)	Case (>25 CL)		**Control (≤25 CL)**	**Case (>25 CL)**	
Sample size, *n* (%)	29 (39.7)	44 (60.3)	-	69 (64.5)	38 (35.5)	-	42 (47.7)	46 (52.3)	-
Age in years, mean (SD)	73.9 (4.3)	76.9 (7.4)	0.0343	73.7 (6.6)	77.4 (8.8)	0.0253	73.6 (13.2)	73.5 (13.1)	ns
Sex (female), *n* (%)	11 (37.9)	22 (50.0)	ns	39 (56.5)	19 (50.0)	ns	18 (42.9)	17 (37.0)	ns
APOE ε4, *n* (%)	7 (24.1)	20 (45.5)	0.0975	13 (18.8)	18 (47.3)	0.0025	6 (14.3)	29 (63.0)	0.0005
Years of education, mean (SD)	12.4 (2.9)	12.3 (2.8)	ns	14.7 (3.4)	13 (3.1)	0.0174	13.7 (3.4)	12.5 (3.1)	ns
Aβ burden, CL mean (SD)	1.7 (13.5)	78.8 (33.1)	<0.0001	2.2 (7.8)	102.1 (41.2)	<0.0001	3.7 (8.3)	96.4 (44.5)	<0.0001
CN, *n* (%)	28 (96.6)	22 (50.0)	-	60 (87.0)	16 (42.1)	-	36 (85.7)	8 (17.4)	-
MCI, *n* (%)	0 (0.0)	15 (34.1)	-	5 (7.2)	9 (26.7)	-	5 (11.9)	16 (34.8)	-
AD, *n* (%)	1 (3.4)	7 (15.9)	-	4 (5.8)	13 (34.2)	-	1 (2.4)	22 (47.8)	-
EM, mean (SD)	0.0 (0.7)	−0.9 (1.3)	0.0006	0.1 (0.9)	−1.3 (1.6)	<0.0001	−0.5 (1.0)	−0.9 (1.6)	ns
Slope of EM, mean (SD)	0.0 (0.1)	−0.1 (0.2)	0.0005	0.1 (0.2)	0.0 (0.2)	0.0176	0.0 (0.1)	−0.1 (0.3)	ns
PACC, mean (SD)	0.0 (0.7)	−0.8 (1.2)	0.0006	0.0 (0.9)	−1.6 (1.6)	<0.0001	−0.1 (0.8)	−1.0 (1.4)	ns
Slope of PACC, mean (SD)	0.0 (0.1)	−0.2 (0.2)	0.0004	0.1 (0.2)	−0.1 (0.2)	0.0003	0.0 (0.1)	−0.1 (0.3)	ns

^1^ Based on *t*-test for continuous variables and chi-square test for categorical variables. Same for all columns named with “*p*”. ^2^ For validating P2X7 results. ^3^ For validating HLA-DR results. ns: no significance.

**Table 2:** Characteristics of sample cohorts with two PRS calculations.

Demographics		AIBL			ADNI			Combined		
		CN	MCI	AD	CN	MCI	AD	CN	MCI	AD
**Sample size**	*n*	647	77	176	255	379	152	902	456	328
**Sex (women)**	*n*	377	40	102	134	154	61	511	194	163
	%	0.583	0.520	0.580	0.526	0.406	0.401	0.567	0.425	0.497
**PRS-pore**	mean	0.293	0.292	0.321	0.243	0.282	0.191	0.279	0.284	0.261
	SD	0.958	1.063	0.984	0.958	1.063	0.984	0.947	0.940	0.962
**PRS-phago**	mean	−0.057	−0.077	−0.054	−0.050	−0.056	−0.069	−0.055	−0.059	−0.061
	SD	0.192	0.240	0.187	0.170	0.207	0.197	0.186	0.213	0.192

## Data Availability

Not applicable.

## Supplementary Materials

The following supporting information can be downloaded at: https://www.mdpi.com/article/10.3390/ijms23147867/s1. References [43,44,45,46,47,48,49,50] were cited in the Supplementary Materials.

## Abbreviations

AD	Alzheimer’s disease
ADNI	Alzheimer’s Disease Neuroimaging Initiative
AIBL	Australian Imaging, Biomarker & Lifestyle Flagship Study of Ageing
Aβ	Beta-amyloid
BBB	Blood-brain barrier
CL	Centiloid
CN	Cognitively normal
CNS	Central nervous system
CR3	Complement receptor 3
CR4	Complement receptor 4
CSF	Cerebrospinal fluid
EM	Episodic memory
GWAS	Genome-wide association studies
MCI	Mild cognitive impairment
MFI	Mean fluorescence intensity
MRI	Magnetic resonance imaging
NF-κB	Nuclear factor kappa light chain enhancer of activated B cells
NK cell	Natural killer cell
P2X7	P2X purinoceptor 7
PACC	Preclinical Alzheimer’s Cognitive Composite
PET	Positron emission tomography
P-tau181P	Tau phosphorylated at threonine 181
SNP	Single nucleotide polymorphism
SP1	Specificity protein 1
T-tau	Total tau
