# Supplementary material for: Identification of Leukocyte Surface P2X7 as a Biomarker Associated with Alzheimer’s Disease
Source: Int J Mol Sci. 2022 Jul 17;23(14):7867. doi: 10.3390/ijms23147867 (PMC9322488; doi:10.3390/ijms23147867)
Supplement: Supplementary file 1 [file ijms-23-07867-s001.zip › ijms-1799323-supplementary.pdf]

Supplementary materials:

Table S1. MFI of P2X7, CD11b, and CD11c on leukocyte subtypes in the discovery cohort (Model 1)[1].

Variable	Discovery cohort (Model 1)		
	**Control (≤ 25 CL[2])**	**Case (> 25 CL)**	**Effect size of Case (> 25 CL)**
P2X7 MFI on total lymphocytes	16.9 ± 4.8	14.1 ± 3.3	-0.572
P2X7 MFI on NK cells ($CD14^-CD16^+$)	20.1 ± 7.4	16.3 ± 5.3	-0.509
P2X7 MFI on B&T cells ($CD14^-CD16^-$)	16.3 ± 4.5	13.8 ± 3.0	-0.559
P2X7 MFI on total monocytes	68.9 ± 37.0	50.4 ± 28.4	-0.501
P2X7 MFI on non-classical monocytes ($CD14^{dim}CD16^+$)	43.2 ± 22.2	32.0 ± 17.9	-0.503
P2X7 MFI on intermediate monocytes ($CD14^+CD16^+$)	82.5 ± 49.0	57.3 ± 34.1	-0.514
P2X7 MFI on classical monocytes ($CD14^+CD16^-$)	72.0 ± 39.7	52.7 ± 29.6	-0.487
P2X7 MFI on total neutrophils	35.1 ± 16.0	25.8 ± 7.9	-0.581
P2X7 MFI on $CD16^{++}$ neutrophils	33.3 ± 13.2	25.0 ± 7.7	-0.625
CD11c MFI on total lymphocytes	9.5 ± 2.6	7.7 ± 2.5	-0.701
CD11c MFI on total monocytes	87.5 ± 38.4	62.9 ± 26.6	-0.639
CD11c MFI on total neutrophils	24.9 ± 10.8	18.6 ± 6.0	-0.580
CD11b MFI on total lymphocytes	48.3 ± 23.6	35.5 ± 18.8	-0.541
CD11b MFI on total monocytes	458.9 ± 214.4	319.2 ± 137.8	-0.651
CD11b MFI on total neutrophils	397.8 ± 182.9	296.1 ± 130.1	-0.556

[1] Data was calculated as mean ± standard deviation. Same for Table S2, S3, and S4.

[2] CL: Centiloid – the scale of PET-Aβ burden in the brain.

Table S2. MFI of P2X7, CD11b, and CD11c on leukocyte subtypes in the discovery cohort (Model 2).

Variable	Discovery cohort (Model 2)				
	CN (≤ 25 CL)	CN (> 25 CL)	Dementia[3]	Effect size of CN (> 25 CL)	Effect size of Dementia[4]
P2X7 MFI on total lymphocytes	17.1 ± 4.7	14.7 ± 3.9	13.4 ± 2.5	-0.601	-0.938
P2X7 MFI on NK cells ($CD14^{-}CD16^{+}$)	20.4 ± 7.4	16.9 ± 5.1	15.6 ± 4.0	-0.550	-0.758
P2X7 MFI on B&T cells ($CD14^{-}CD16^{-}$)	16.5 ± 4.5	14.4 ± 3.3	13.1 ± 2.3	-0.615	-0.991
P2X7 MFI on total monocytes	70.6 ± 36.6	53.2 ± 28.9	46.6 ± 21.8	-0.499	-0.689
P2X7 MFI on non-classical monocytes ($CD14^{dim}CD16^{+}$)	44.1 ± 22.1	32.9 ± 17.2	30.6 ± 15.8	-0.560	-0.673
P2X7 MFI on intermediate monocytes ($CD14^{+}CD16^{+}$)	84.6 ± 48.7	58.4 ± 34.5	54.9 ± 29.1	-0.668	-0.758
P2X7 MFI on classical monocytes ($CD14^{+}CD16^{-}$)	73.8 ± 39.3	53.9 ± 29.0	50.1 ± 23.7	-0.564	-0.672
P2X7 MFI on total neutrophils	35.6 ± 16.0	27.9 ± 7.1	23.6 ± 3.4	-0.746	-1.171
P2X7 MFI on $CD16^{++}$ neutrophils	33.7 ± 13.2	27.0 ± 7.3	23.0 ± 3.3	-0.670	-1.067
CD11c MFI on total lymphocytes	9.6 ± 2.6	7.9 ± 2.5	7.5 ± 2.4	-0.672	-0.831
CD11c MFI on total monocytes	88.5 ± 38.9	65.1 ± 26.5	60.8 ± 29.9	-1.054	-1.249
CD11c MFI on total neutrophils	24.9 ± 11.0	20.3 ± 6.9	17.3 ± 6.3	-0.845	-1.405
CD11b MFI on total lymphocytes	49.2 ± 23.5	35.1 ± 18.3	35.3 ± 19.3	-0.767	-0.755
CD11b MFI on total monocytes	465.0 ± 216.2	326.7 ± 137.9	312.2 ± 152.7	-1.186	-1.311
CD11b MFI on total neutrophils	396.4 ± 186.3	302.8 ± 130.8	296.7 ± 143.6	-0.808	-0.861

[3] Dementia: patients classified with MCI +ve and AD.

[4] Effect size of dementia compared with CN (CL ≤ 25).

Table S3. MFI of P2X7 on leukocyte subtypes in the validation cohort A (Model 1).

Variable	Validation A (Model 1)		
	**Control (≤ 25 CL)**	**Case (> 25 CL)**	**Effect size of Case (> 25 CL)**
P2X7 MFI on total lymphocytes	4.4 ± 1.4	4 ± 0.8	-0.381
P2X7 MFI on NK cells ($CD14^{-}CD16^{+}$)	13.0 ± 21.6	8.6 ± 8.3	-0.535
P2X7 MFI on B&T cells ($CD14^{-}CD16^{-}$)	3.7 ± 0.5	3.6 ± 0.4	-0.319
P2X7 MFI on total monocytes	13.7 ± 10.3	11 ± 4.5	-0.573
P2X7 MFI on intermediate monocytes ($CD14^{+}CD16^{+}$)	12.8 ± 4.9	11.9 ± 5.0	-0.174
P2X7 MFI on classical monocytes ($CD14^{+}CD16^{-}$)	10.1 ± 2.5	9.4 ± 2.5	-0.285
P2X7 MFI on non-classical monocytes ($CD14^{dim}CD16^{+}$)	14.7 ± 13.6	9.1 ± 4.5	-1.233
P2X7 MFI on total neutrophils	9.3 ± 4.0	8 ± 0.7	-0.788
P2X7 MFI on $CD16^{++}$ neutrophils	9.3 ± 4.9	8.4 ± 3.1	-0.188

Table S4. MFI of P2X7 on leukocyte subtypes in the validation cohort A (Model 2).

Variable	Validation cohort (Model 2)					
	CN (≤ 25 CL)	CN (> 25 CL)	Effect size of CN (> 25 CL)[5]	MCI (≤ 25 CL)	Dementia[6]	Effect size of Dementia[7]
P2X7 MFI on total lymphocytes	4.4 ± 1.5	3.8 ± 0.6	-0.997	4.6 ± 0.9	4.1 ± 0.9	-0.566
P2X7 MFI on NK cells ($CD14^-CD16^+$)	13.8 ± 23.0	6.2 ± 6.1	-1.234	10.0 ± 7.5	9.4 ± 8.8	-0.082
P2X7 MFI on B&T cells ($CD14^-CD16^-$)	3.7 ± 0.5	3.6 ± 0.5	-0.228	4.0 ± 0.4	3.5 ± 0.4	-1.175
P2X7 MFI on total monocytes	14.0 ± 10.8	11.1 ± 5.6	-0.522	14.2 ± 6.7	10.6 ± 3.7	-0.530
P2X7 MFI on intermediate monocytes ($CD14^+CD16^+$)	13.0 ± 5.2	11.5 ± 3.2	-0.480	12.3 ± 1.6	12.0 ± 5.7	-0.250
P2X7 MFI on classical monocytes ($CD14^+CD16^-$)	10.3 ± 2.5	10.0 ± 2.8	-0.093	10.0 ± 1.2	8.9 ± 2.2	-0.933
P2X7 MFI on non-classical monocytes ($CD14^-CD16^+$)	15.1 ± 14.1	8.9 ± 5.3	-1.183	14.8 ± 12.3	9.0 ± 3.8	-0.473
P2X7 MFI on total neutrophils	9.3 ± 4.0	7.6 ± 1.4	-1.204	8.2 ± 1.6	8.6 ± 3.2	0.224
P2X7 MFI on $CD16^{++}$ neutrophils	9.3 ± 5.0	7.3 ± 1.4	-1.477	8.1 ± 1.5	8.5 ± 3.4	0.262

[5] Effect size of CN (> 25 CL) compared with CN (≤ 25 CL).
[6] Dementia: patients classified with MCI +ve and AD.
[7] Effect size of dementia compared with MCI (CL ≤ 25).

Table S5. 12 P2X7 and P2X4 SNPs and their corresponding functional scores.

CHR	SNP	AA	Gene	PRS-pore[8]				PRS-phago[9]				Reference
				function	0EA	1EA	2EA	function	0EA	1EA	2EA	
12	rs17525809	V76A	P2X7	LOF	0	-0.5	-1	No change	0	0	0	[43]
12	rs28360447	G150R	P2X7	LOF	0	-0.5	-1	LOF	0	-0.25	-0.5	[44]; [45]
12	rs208294	H155Y[10]	P2X7	GOF	0	0.5	1	No change	0	0	0	[45]; [46]
12	rs7958311	R270H	P2X7	LOF	0	-0.25	-0.5	No change	0	0	0	[43]; [45]
12	rs7958316	R276H	P2X7	LOF	0	-0.5	-1	No change	0	0	0	[43]; [45]
12	rs28360457	R307Q	P2X7	LOF	0	-1	-1	Change	0	0	0	[45]; [47]
12	rs1718119	A348T[11]	P2X7	GOF	0	0.5	1	No change	0	0	0	[45]; [46]
12	rs2230911	T357S	P2X7	LOF	0	-0.5	-1	No change	0	0	0	[43]
12	rs2230912	Q460R	P2X7	No Change	0	0	0	No change	0	0	0	[45]; [46]
12	rs3751143	E496A	P2X7	LOF	0	-0.5	-1	No change	0	0	0	[48]; [49]
12	rs1653624	I568N	P2X7	LOF	0	-0.75	-1	LOF	0	-0.5	-1	[45]; [50]
12	rs28360472	Y315C	P2X4	No change	0	0	0	LOF	0	-0.8	-1	[44]

[8] To assess the pore formation function of P2X7

[9] To assess the innage phagocytic function of P2X7

[10] If both H155Y and A348T were heterozygous, PRS-pore added 0.5; if one was germline and another was homozygous, PRS-pore added 0.75; if both were homozygous, PRS-pore added 1.

[11] If both H155Y and A348T were heterozygous, PRS-pore added 0.5; if one was germline and another was homozygous, PRS-pore added 0.75; if both were homozygous, PRS-pore added 1.

Table S6. 12 P2X7 and P2X4 SNPs in the AIBL cohort (647 CN, 77 MCI, and 176 AD).

CHR	SNP	AA	Gene	A1	A2	F_A	F_U	CHISQ	P	OR	MAF_CN[12]	MAF_MCI[13]	MAF_AD[14]
12	rs17525809	**V76A**	P2X7	C	T	0.072	0.099	3.858	**0.050**	0.702	0.072	0.087	0.072
12	rs28360447	G150R	P2X7	A	G	0.014	0.025	2.760	0.097	0.548	0.014	0.040	0.014
12	rs208294	H155Y	P2X7	T	C	0.434	0.440	0.070	0.791	0.973	0.434	0.427	0.434
12	rs7958311	R270H	P2X7	A	G	0.274	0.260	0.403	0.525	1.077	0.274	0.286	0.274
12	rs7958316	R276H	P2X7	A	G	0.027	0.025	0.092	0.761	1.105	0.027	0.048	0.027
12	rs28360457	R307Q	P2X7	A	G	0.017	0.019	0.126	0.723	0.873	0.017	0.024	0.017
12	rs1718119	A348T	P2X7	T	C	0.389	0.414	1.010	0.315	0.900	0.389	0.389	0.389
12	rs2230911	T357S	P2X7	G	C	0.075	0.073	0.047	0.829	1.044	0.075	0.063	0.075
12	rs2230912	Q460R	P2X7	G	A	0.177	0.164	0.451	0.502	1.097	0.177	0.175	0.161
12	rs3751143	E496A	P2X7	G	T	0.170	0.172	0.009	0.925	0.987	0.170	0.167	0.170
12	rs1653624	I568N	P2X7	A	T	0.029	0.021	0.894	0.344	1.386	0.029	0.032	0.029
12	rs28360472	Y315C	P2X4	G	A	0.013	0.015	0.150	0.698	0.846	0.013	0.024	0.013

[12] Minor allele frequency (MAF) in the CN cohort.
[13] MAF in the MCI cohort.
[14] MAF in the AD cohort.

Table S7. 12 P2X7 and P2X4 SNPs in the ADNI cohort (255 CN, 379 MCI, and 152 AD).

CHR	SNP	AA	Gene	A1	A2	F_A	F_U	CHISQ	P	OR	MAF_CN	MAF_MCI	MAF_AD
12	rs17525809	V76A	P2X7	C	T	0.081	0.073	0.193	0.660	1.123	0.073	0.065	0.081
12	rs28360447	G150R	P2X7	A	G	0.016	0.019	0.145	0.704	0.811	0.019	0.014	0.016
12	rs208294	H155Y	P2X7	T	C	0.438	0.437	0.001	0.980	1.004	0.437	0.447	0.438
12	rs7958311	R270H	P2X7	A	G	0.255	0.286	1.001	0.317	0.852	0.286	0.245	0.255
12	rs7958316	R276H	P2X7	A	G	0.016	0.023	0.551	0.458	0.673	0.023	0.017	0.016
12	rs28360457	R307Q	P2X7	A	G	0.025	0.021	0.135	0.714	1.188	0.021	0.009	0.025
12	rs1718119	A348T	P2X7	T	C	0.370	0.361	0.068	0.794	1.039	0.361	0.372	0.370
12	rs2230911	T357S	P2X7	G	C	0.087	0.082	0.062	0.803	1.065	0.082	0.076	0.087
12	rs2230912	Q460R	P2X7	G	A	0.154	0.170	0.911	0.34	0.891	0.153	0.140	0.146
12	rs3751143	E496A	P2X7	C	A	0.186	0.174	0.218	0.640	1.090	0.174	0.188	0.186
12	rs1653624	I568N	P2X7	A	T	0.034	0.021	1.366	0.243	1.650	0.021	0.025	0.034
12	rs28360472	Y315C	P2X4	G	A	0.016	0.011	0.258	0.611	1.362	0.011	0.014	0.016

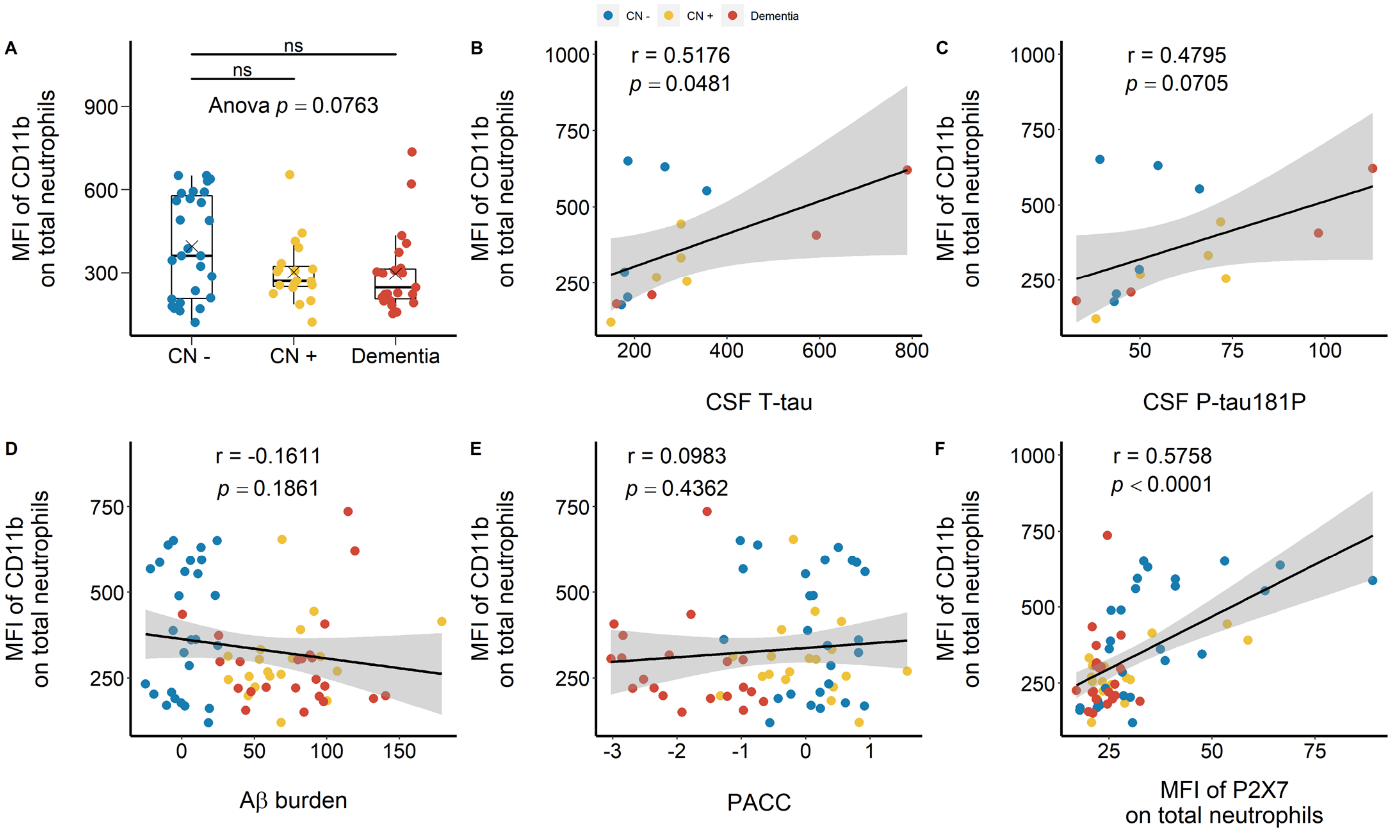

Figure S1. CD11b expression on peripheral neutrophils in the discovery cohort. **A.** CD11b expressions on total neutrophils between CN (CN -: CN -ve), pre-clinical (CN +: CN +ve), and AD with dementia (dementia: MCI +ve and AD) individuals. Bar graphs illustrated the boxplot distribution of individual measurements with "x" denoting the mean. Three-group comparison was determined by one-way ANOVA followed by multiple comparison using Dunnett's post-hoc test (solid line). **B-E.** The associations between neutrophils CD11b expressions, CSF biomarkers (μg/L), Aβ burden (CL) measured by PET, and PACC. **F.** Association between neutrophil CD11b expression and neutrophil P2X7 expression. Correlation r and P values were calculated by Pearson product-moment correlational analysis. The "grey" band indicated the 95% confidence interval of the black linear regression line. ns: no significance.

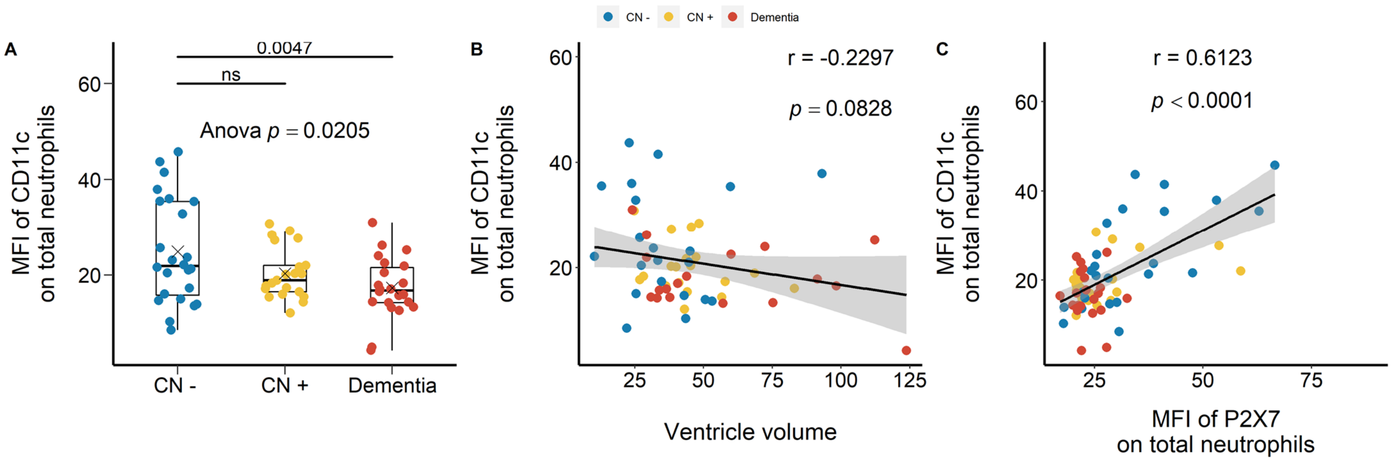

Figure S2. CD11c expression on peripheral neutrophils in the discovery cohort. **A.** CD11c expressions on total neutrophils between CN (CN -: CN -ve), pre-clinical (CN +: CN +ve), and AD with dementia (dementia: MCI +ve and AD) individuals. Bar graphs illustrated the boxplot distribution of individual measurements with "x" denoting the mean. Three-group comparison was determined by one-way ANOVA followed by multiple comparison using Dunnett's post-hoc test (solid line). **B-C.** The associations between neutrophil CD11c expressions, ventricle volume, and neutrophil P2X7 expression. Correlation r and P values were calculated by Pearson product-moment correlational analysis. The "grey" band indicated the 95% confidence interval of the black linear regression line. ns: no significance.

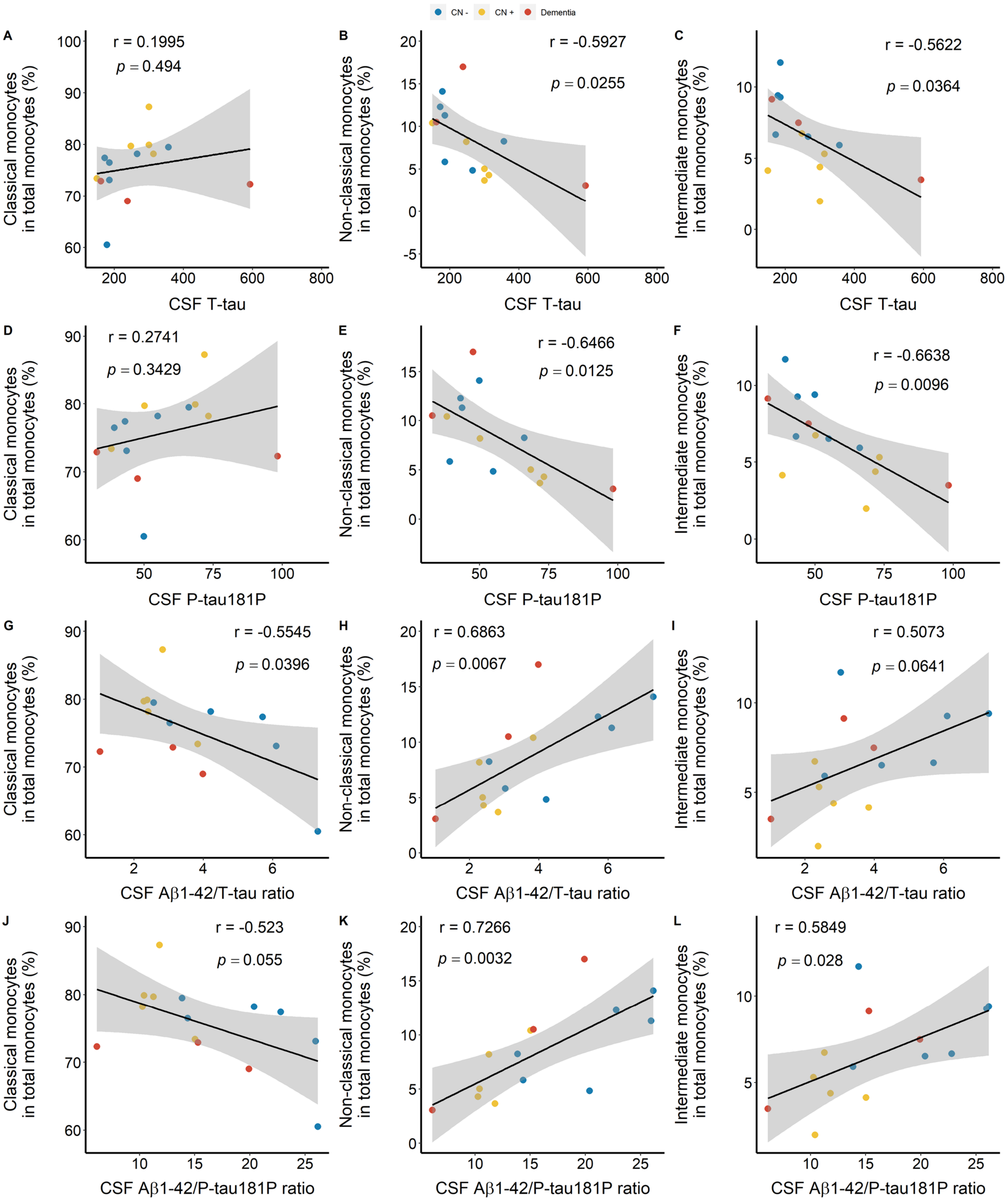

Figure S3. Monocyte subpopulations associated with CSF biomarkers in the discovery cohort. **A-C.** Associations with CSF T-tau concentration (μg/L). **D-F.** Associations with CSF P-tau181P concentration (μg/L). **G-I.** Associations with CSF Aβ_{1-42}/T-tau ratio. **J-L.** Associations with CSF Aβ_{1-42}/P-tau181P ratio. Correlation r and P values were calculated by Pearson product-moment correlational analysis. The "grey" band indicated the 95% confidence interval of the black linear regression line.

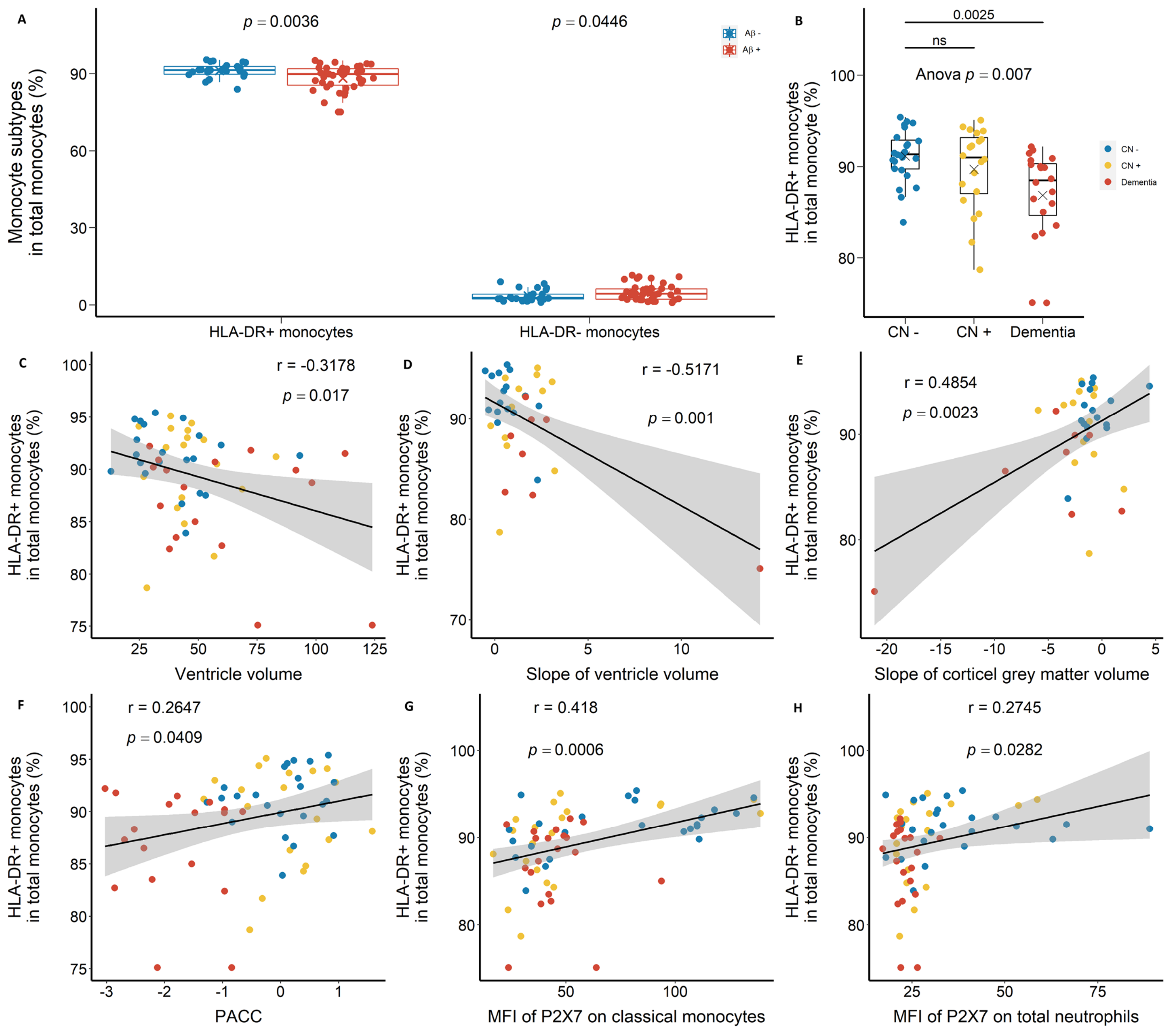

Figure S4. Relative percentages of HLA-DR⁺ and HLA-DR⁻ monocytes in total monocytes in the discovery cohort. **A.** Comparisons of HLA-DR+ and HLA-DR- monocytes between Aβ +ve cases (Aβ +) and Aβ -ve controls (Aβ -). Bar graphs illustrated the boxplot distribution of individual measurements with "x" de-noting the mean. Two-group comparison was determined by *t*-test. **B.** Comparison of HLA-DR⁺ monocytes between CN (CN –: CN -ve), pre-clinical (CN +: CN +ve), and AD with dementia individuals (dementia: MCI +ve and AD). Bar graphs illustrated the boxplot distribution of individual measurements with "x" de-noting the mean. Three-group comparison was determined by one-way ANOVA followed by multiple comparison using Dunnett's post-hoc test (solid line). **C-H.** Associations between HLA-DR⁺ monocytes, brain atrophy, PACC, and P2X7 expressions. Correlation r and P values were calculated by Pearson product-moment correlational analysis. The "grey" band indicated the 95% confidence interval of the black linear regression line. ns: no significance.

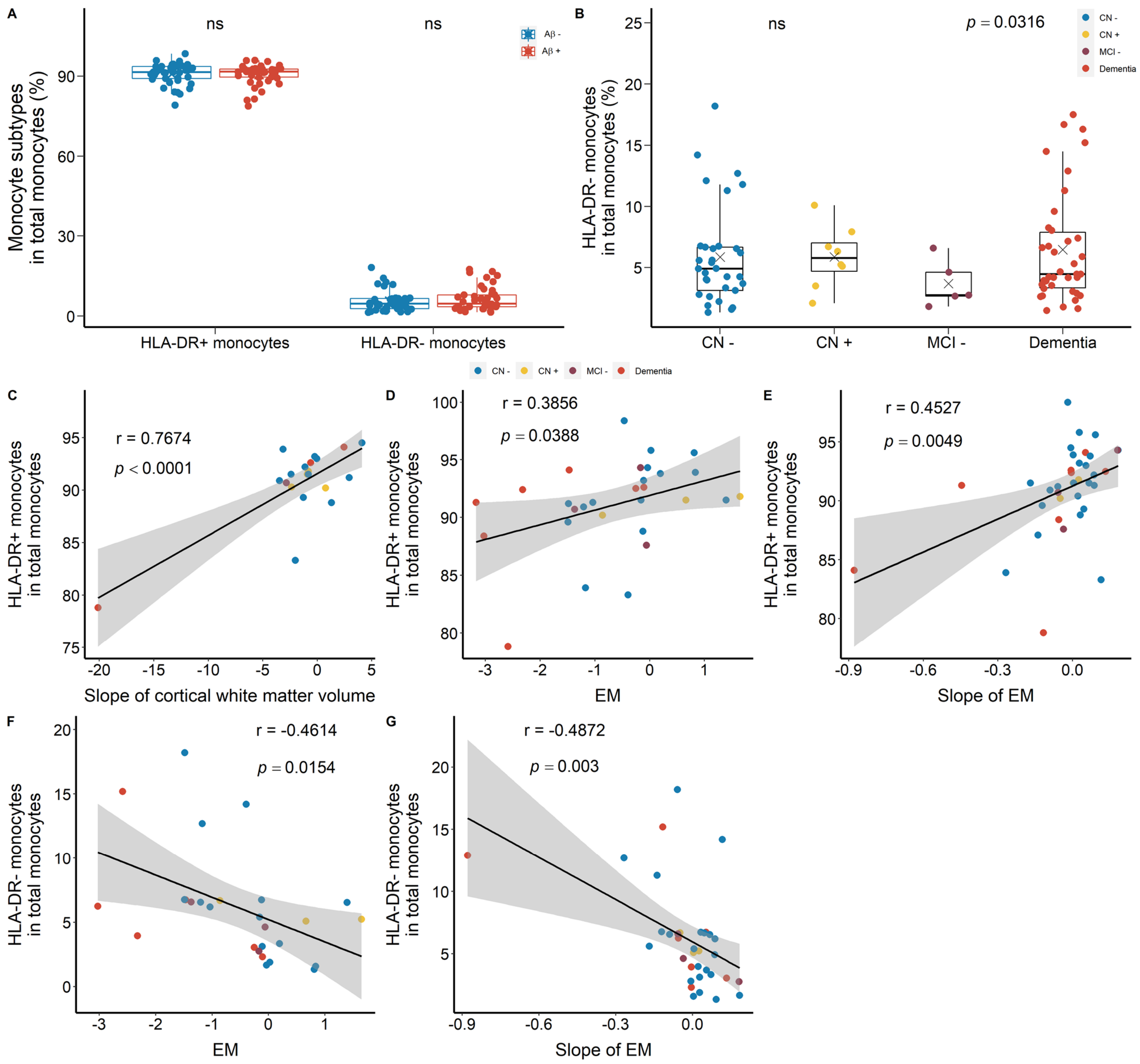

Figure S5. Relative percentages of HLA-DR$^+$ and HLA-DR$^-$ monocytes in total monocytes in the validation cohort B. **A.** Comparisons of HLA-DR$^+$ and HLA-DR$^-$ monocytes between Aβ +ve cases (Aβ +) and Aβ -ve controls (Aβ -). **B.** Comparison of HLA-DR$^-$ monocytes between CN (CN -: CN -ve) and pre-clinical (CN +: CN +ve) individuals. Comparison of HLA-DR$^-$ monocytes between prodromal (MCI -: MCI -ve) and AD with dementia individuals (dementia: MCI +ve and AD). Bar graphs illustrated the boxplot distribution of individual measurements with "x" denoting the mean. Two-group comparison was determined by *t*-test. **C-G.** Associations between HLA-DR$^+$ monocytes, brain atrophy and EM. Correlation r and P values were calculated by Pearson product-moment correlational analysis. The "grey" band indicated the 95% confidence interval of the black linear regression line. ns: no significance.
